# Molecular analysis of single circulating tumour cells following long‐term storage of clinical samples

**DOI:** 10.1002/1878-0261.12113

**Published:** 2017-10-24

**Authors:** Barbara Mesquita, Dominic G. Rothwell, Deborah J Burt, Francesca Chemi, Fabiola Fernandez‐Gutierrez, Daniel Slane‐Tan, Jenny Antonello, Mathew Carter, Louise Carter, Marina Parry, Lynsey Franklin, Richard Marais, Fiona Blackhall, Caroline Dive, Ged Brady

**Affiliations:** ^1^ Clinical & Experimental Pharmacology Group CRUK Manchester Institute University of Manchester UK; ^2^ Division of Molecular and Clinical Cancer Sciences University of Manchester UK; ^3^ Molecular Oncology Group CRUK Manchester Institute University of Manchester UK; ^4^ Institute of Cancer Sciences University of Manchester UK; ^5^ Cancer Research UK Lung Cancer Centre of Excellence UK

**Keywords:** circulating tumour cells, molecular analysis, single cells, stability, storage

## Abstract

The CellSearch^®^ semiautomated CTC enrichment and staining system has been established as the ‘gold standard’ for CTC enumeration with CellSearch^®^ CTC counts recognized by the FDA as prognostic for a number of cancers. We and others have gone on to show that molecular analysis of CellSearch^®^ CTCs isolated shortly after CellSearch^®^ enrichment provides another valuable layer of information that has potential clinical utility including predicting response to treatment. Although CellSearch^®^ CTCs can be readily isolated after enrichment, the process of analysing a single CellSearch^®^ patient sample, which may contain many CTCs, is both time‐consuming and costly. Here, we describe a simple process that will allow storage of all CellSearch^®^‐enriched cells in glycerol at −20 °C for up to 2 years without any measurable loss in the ability to retrieve single cells or in the genome integrity of the isolated cells. To establish the suitability of long‐term glycerol storage for single‐cell molecular analysis, we isolated individual CellSearch^®^‐enriched cells by DEPArray™ either shortly after CellSearch^®^ enrichment or following storage of matched enriched cells in glycerol at −20 °C. All isolated cells were subjected to whole‐genome amplification (WGA), and the efficacy of single‐cell WGA was evaluated by multiplex PCR to generate a Genome Integrity Index (GII). The GII results from 409 single cells obtained from both ‘spike‐in’ controls and clinical samples showed no statistical difference between values obtained pre‐ and postglycerol storage and that there is no further loss in integrity when DEPArray™‐isolated cells are then stored at −80 °C for up to 2 years. In summary, we have established simple yet effective ‘stop‐off’ points along the CTC workflow enabling CTC banking and facilitating selection of suitable samples for intensive analysis once patient outcomes are known.

**Abbreviations**
CTC: circulating tumour cell
SD: standard deviation
TC: tumour cell
WBC: white blood cell
WGA: whole‐genome amplification

## 1 Introduction

Molecular analysis of tumours has led to the identification of mutational drivers of cancer and the development of targeted therapies, which when used to treat stratified patient cohorts have been shown to lead to significant improved response rates (Sharma *et al*., 2007; Sosman *et al*., 2012; Vogel *et al*., 2002). The molecular characterization of tumours also has the potential to give insight into mechanisms of resistance to therapies and tumour heterogeneity (Burrell and Swanton, 2014; Carter *et al*., 2017; Casasent *et al*., 2017; Jamal‐Hanjani *et al*., 2015; Navin, 2015). Circulating tumour cells (CTCs) found in the blood of patients with cancer are being evaluated for clinical utility as a liquid biopsy that can facilitate molecular characterization of the patient's disease (Carter *et al*., 2017; Diaz and Bardelli, 2014; Fusi *et al*., 2013; Krebs *et al*., 2014; Rothwell *et al*., 2016). Use of a simple blood draw and CTC analysis has the added benefit of ease of sample collection as well as the possibility of repeat sampling. CTC number is associated with prognosis, response to chemotherapy and identification of disease recurrence in several types of cancer including lung, colorectal, breast and prostate (Attard and de Bono, 2011; de Bono *et al*., 2008; Cohen *et al*., 2008; Cristofanilli *et al*., 2004; Crosbie *et al*., 2016; Krebs *et al*., 2011; Miller *et al*., 2010; Rack *et al*., 2014; Smerage *et al*., 2014; Vargas and Harris, 2016). Recently, CTCs have been used to infer the genetic status of the patient's tumour and to identify a molecular profile linked to response to therapy (Carter *et al*., 2017; Lowes *et al*., 2015; Neal and Lilja, 2016). Downstream molecular analysis of isolated CTCs can also give greater insight into intratumour heterogeneity and identify specific signalling pathways that could be explored in the clinical setting.

However, detailed analysis of CTCs presents difficulties; the paucity of CTCs in blood, associated enriching processes and downstream molecular analyses are technically challenging and expensive. A variety of technologies have been developed to enrich CTCs that commonly rely on cell surface antigen expression or on physical properties including cell size and deformability that distinguish CTCs from blood cells (Alix‐Panabières and Pantel, 2014; Alix‐Panabières and Pantel, 2016; Chudziak *et al*., 2016; Ferreira *et al*., 2016; Pantel, 2016). CellSearch^®^ is a FDA‐approved epitope‐dependent platform capable of CTC enrichment and enumeration. To enable single‐cell analysis, enriched samples require further manipulation on platforms such as the DEPArray™ (Silicon Biosystems, Bologna, Italy), a microfluidic system that combines high‐resolution imaging with the dielectrophoretic movement of fluorochrome‐labelled cells enabling selection as well as isolation of individual cells. We and others demonstrated the utility of a combined CellSearch^®^ and DEPArray™ workflow to enable molecular analysis of CTCs (Carter *et al*., 2017; De Luca *et al*., 2016; Hodgkinson *et al*., 2014). Although effective, the isolation of CTCs using the combined CellSearch^®^ and DEPArray™ approach is both time‐consuming and costly, making it impractical for routine analysis of all CellSearch^®^‐enriched samples. Furthermore, it is often not clear which clinical samples will warrant CTC isolation and detailed molecular analysis. For example, in the course of a project or clinical trial, it may be that all pretreatment samples are collected but only a subset will be of interest based on a patient's response to treatment.

**Figure 1:**
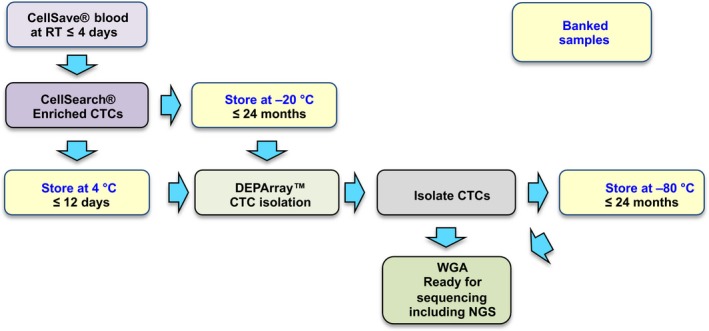
Schematic showing the workflow developed for the isolation, storage and genomic analysis of circulating tumour cells (CTCs) from clinical blood samples. Yellow boxes with blue text indicate stop‐off points where material can be stored prior to downstream processing.

To make the CellSearch^®^ and DEPArray™ approach more flexible and compatible with clinical trials, we have established a simple yet robust workflow (Fig. 1), which allows banking of either the entire CellSearch^®^‐enriched sample prior to cell isolation, or individual CTCs after DEPArray™ single‐cell isolation. We show that banked samples can be stored for over a year without any adverse impact on single‐cell whole‐genome amplification (WGA) and subsequent molecular analysis.

## 2 Materials and methods

### 2.1 Blood collection

Blood samples were collected in CellSave^®^ vacutainers and transferred to the Clinical and Experimental Pharmacology laboratory for processing within four days of blood draw. All samples were collected following receipt of informed consent in compliance with the approval from the local Research Ethics Committee [REC reference 07/H1014/96; REC reference 13/LO/1546; MCRC Biobank Research Tissue Bank Ethics (ref: 07/H1003/161 + 5)].

### 2.2 Cell culture

Human colon carcinoma cell line LS174‐T was obtained from the ATCC collection and maintained as recommended. Briefly, LS174‐T cells were maintained in RPMI media, supplemented with 10% (v/v) fetal bovine serum (FBS), penicillin (100 U·mL^−1^) and streptomycin (100 U·mL^−1^). Cells were maintained in a humid atmosphere at 37 °C with 5% CO_2_. Cell survival and counting were determined by a trypan blue exclusion assay.

One thousand tumour cells (TCs) were spiked into a healthy normal volunteer (HNV) 7.5 mL of blood collected in CellSave tubes.

### 2.3 CellSearch^®^ Enrichment

Circulating tumour cells enumeration was performed on a CellSearch^®^ system (Menarini Silicon Biosystems, Inc., San Diego, CA, USA) using the CellSearch^®^ Epithelial Cell Kit (Menarini Silicon Biosystems, Inc). In brief, 7.5 mL of blood was collected in a CellSave preservative vacutainer and stored at room temperature (RT) for up to 96 h. Afterwards, the blood samples were processed into the CellTracks^®^ AutoPrep^®^ system, the CellSearch^®^ Circulating Tumour Cell Kit and the CellTracks^®^ Analyser II (Menarini Silicon Biosystems, Inc.). During the automated CellSearch^®^ process, TCs or CTCs were immunomagnetically captured by anti‐epithelial cell adhesion molecule (EpCAM) antibody‐coated ferrofluid and detected by immunofluorescent staining of pan‐cytokeratins and 4′,6‐diamidino‐2‐phenylindole (DAPI), as well as by negativity for the leucocyte‐specific antigen CD45. TCs and CTCs were identified as DAPI+/CK+/CD45‐ cells, while white blood cells (WBCs) as DAPI+/CK−/CD45+. After scanning, the cartridges were removed from the MagNest™ device and stored at 4 °C in the dark until further processing.

### 2.4 Glycerol storage

Following CellSearch^®^ enrichment, samples with cell counting results between 120 and 200 cells were resuspended, and half of the volume of the CellSearch^®^ cartridge was stored at −20 °C in 50% glycerol prior to isolation using DEPArray™. If the cell count was ≥ 200, one‐third of the resuspended cell solution was stored at 4 °C, one‐third at −20 °C in glycerol and the remaining isolated by DEPArray™.

### 2.5 DEPArray™ isolation

Circulating tumour cells and WBCs prestained with antibodies to CD45 and pan‐CK and DAPI were aspirated from the CellSearch^®^ cartridge used for the CTC enumeration, and single cells were isolated using the DEPArray™ system (Silicon Biosystems) as per the manufacturer's instructions. After CTC enrichment, for samples with CTC counts higher than 5 and less than 120, individual or pooled CTCs and WBCs were isolated by DEPArray™ and immediately stored at −80 °C. If the cell count was higher than 120, the samples were prepared as described in the previous section (2.4 Glycerol Storage). Individual and pools of TCs, CTCs and control WBCs were isolated from samples pre‐ and postglycerol storage. Cells showing positive staining for pan‐cytokeratin (CK), undetectable CD45 labelling and positive nuclear staining were classified as TCs or CTCs, and WBCs were detected as DAPI+/CK−/CD45+. All were subjected to whole‐genome amplification.

For calculation of the number of cells loaded onto the DEPArray™, a correction of the CellSearch^®^ count for each sample evaluated pre‐ and postglycerol storage was performed. This involved reducing the CellSearch^®^ count by 30% to account for loss of sample associated with the dead volume in the DEPArray™ cartridge. The number of aliquots for each sample was then also corrected for to give a final ‘expected DEPArray count’ for CTC and total cell counts (Table 2).

### 2.6 Whole‐genome amplification

Whole‐genome amplification of single or pools of TCs, CTCs and WBCs was performed using the ampli1™ wga kit versions 1 and 2 (Silicon Biosystems), following the manufacturer's instructions to obtain a 50 μL of WGA product. The ampli1™ wga procedure (Silicon Biosystem, Inc.) was performed in a single tube according to the manufacturer's protocol. This whole‐genome amplification method is based on adaptor ligation‐mediated amplification. After cells lysis, the genomic DNA was digested with *Mse*I restriction enzyme to generate sticky end fragments, followed by ligation of a single adaptor on both overhangs of each fragment, and a fill‐in reaction to complement the sequence of the adaptor. The resultant WGA PCR product (50 μL) was produced by amplification of the entire genome library with one single high‐specific PCR primer corresponding to the adaptor.

### 2.7 Genome Integrity Index

The efficacy of WGA was evaluated by a multiplex quality control PCR (GII‐PCR). Concisely, a four‐amplicon multiplex PCR to assess fragments of various lengths in different chromosomes (12p:91 bp; 5p:108–166 bp; 17p:299 bp; and 6p:614 bp) was performed and visualized on a 1.5% (w/v) agarose gel. This quality control step allowed us to establish a Genome Integrity Index (GII) of 0–4 for each sample.

It has been observed that the number of bands amplified (0–4) is associated with the integrity of the starting material, with cells with good quality DNA presenting 3 or 4 PCR bands, while cells with degraded DNA present fewer bands (Polzer *et al*., 2014). For the mean GII values, the GII value for each individual single cell was determined and used to determine the mean for the entire sample. Human genomic DNA was used as a positive control.

### 2.8 Statistical analysis

Tests to evaluate the normality of each group of data were performed (D′Agostino–Pearson and Shapiro–Wilk), and accordingly, statistical tests were further performed. Comparisons within and across groups were made by Mann–Whitney, *t*‐test, Kruskal–Wallis, Wilcoxon, and Friedman's tests (with Dunn's correction). *P* values < 0.05 were classified as significant.

## 3 Results

**Figure 2:**
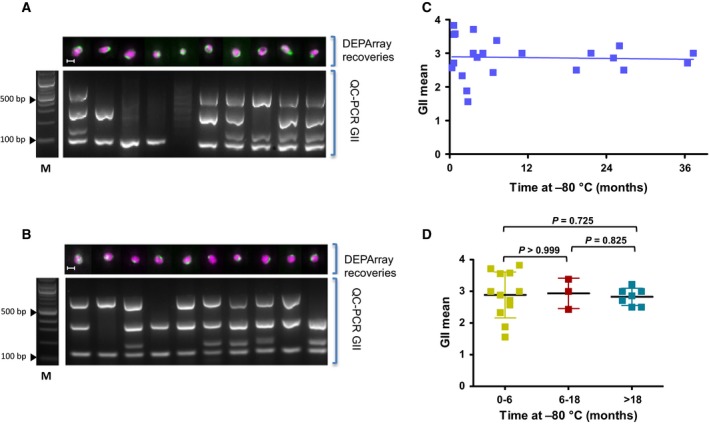
(A) Molecular analysis of isolated SCLC CTCs following short‐term storage. After CellSearch^®^ enrichment and DEPArray™ isolation, cells were stored at −80 °C for 11 days. All cells underwent WGA and GII‐PCR to determine their Genome Integrity Index (GII). The top panel shows DEPArray images of cells labelled with CK (green) and DAPI (pink) with the scale bar representing 10 μm. The bottom panel shows an agarose gel of the GII‐PCR products with 0–4 bands generated for four different genomic regions with different sizes (12p:91 bp; 5p:108–166 bp; 17p:299 bp; and 6p:614 bp). *M* = 100‐bp ladder. (B) Molecular analysis of CTCs isolated from patients with SCLC after enrichment by CellSearch^®^, isolation by DEPArray™ and storage at −80 °C for 25 months. Isolated cells then underwent WGA and GII‐PCR to determine GII and data presented using the same format as in panel A. (C) Graphical representation of the mean GII of CTCs following storage at −80 °C for up to 36 months for 22 patients with SCLC. Linear regression analysis found no detrimental effect of long‐term storage on GII (blue trend line) (*P* = 0.8429; *R* square = 0.002011). (D) Graphical representation of GII mean value grouped according to time stored at −80 °C following DEPArray™ isolation. No statistically significant difference was seen across all groups (Mann–Whitney test, *P* values >0.05; error bars show SD).

We and others have previously demonstrated the potential clinical benefit of molecular characterization of CTCs following single‐cell isolation from patient blood samples (Carter *et al*., 2017; Diaz and Bardelli, 2014; Fusi *et al*., 2013; Rothwell *et al*., 2016). To facilitate single CTC isolation and analysis, we have established a simple yet robust workflow, which allows banking of either the entire CellSearch^®^ sample prior to cell isolation, or individual CTCs after DEPArray™ single‐cell isolation (Fig. 1). To address the stability of stored samples, we first compared the effect of long‐term storage at −80 °C on CTCs which had undergone CellSearch^®^ enrichment, followed by DEPArray™ isolation. We examined CTCs from 22 small cell lung cancer (SCLC) patient samples that had undergone CellSearch^®^ enrichment, followed by DEPArray™ isolation (Fig. 1; Table 1). Following storage at −80 °C from 11 days (0.4 months) to 37 months, DEPArray™ CTCs were subjected to WGA using ampli1™ followed by a GII‐PCR quality check for each cell (Fig. 2A,B). Comparable GII values were seen in all isolated single cells, regardless of time stored at −80 °C, and no observable or statistical drop in GII was seen over time (Fig. 2C,D). These data demonstrate that isolated single CTCs can be reliably stored at −80 °C for up to 37 months with no significant detrimental effect on the genetic integrity of the cells.

**Table 1:** Characterization of the 22 clinical samples from small cell lung cancer (SCLC) patients detailing the number of CTCs after CellSearch^®^ enumeration. For each patient the time that DEPArray isolated CTCs were stored at −80 °C prior to WGA is shown in months

Patient	Disease type	CellSearch^®^ Count	Time at −80 °C (months)
1	SCLC	222	37.0
2	SCLC	23 243	36.0
3	SCLC	7687	27.0
4	SCLC	2048	26.0
5	SCLC	44	25.0
6	SCLC	370	22.0
7	SCLC	377	19.0
8	SCLC	4061	11.0
9	SCLC	669	7.0
10	SCLC	109	7.0
11	SCLC	237	5.0
12	SCLC	35	4.0
13	SCLC	58	4.0
14	SCLC	1187	4.0
15	SCLC	11	3.0
16	SCLC	43	3.0
17	SCLC	836	2.0
18	SCLC	3780	0.9
19	SCLC	207	0.7
20	SCLC	1018	0.6
21	SCLC	1173	0.6
22	SCLC	381	0.4

**Figure 3:**
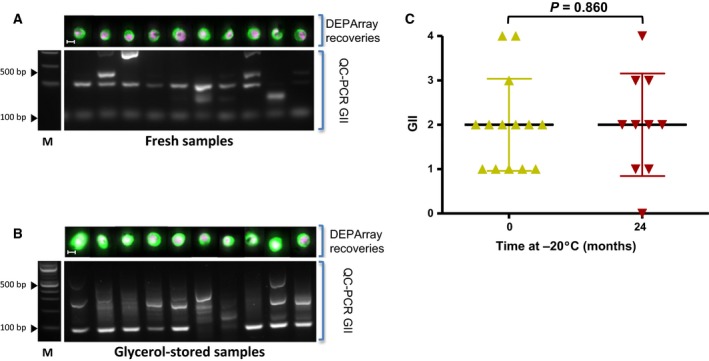
(A) Cultured LS174T cells were added to HNV blood, enriched by CellSearch^®^ and isolated by DEPArray™. All cells underwent WGA and GII‐PCR to determine the GII and data presented using the same format as in Fig. 2 panel A. (B) LS174T cells recovered from the CellSearch^®^ cartridge and stored at −20 °C in glycerol for 24 months prior to DEPArray™ isolation. Following WGA, GII‐PCR was used to determine GII for each sample and data presented using the same format as in Fig. 2 panel A. (C) A comparison of the GII of individual cells enriched by CellSearch^®^ and DEPArray™ isolated prior to glycerol storage (0 months) and stored at −20 °C for 24 months in glycerol before DEPArray™. No statistically significant difference (Mann–Whitney test; *P* = 0.860; error bars show SD) was seen.

To extend the flexibility of our sample processing workflow (Fig. 1), we next tested whether cells could be stored post‐CellSearch^®^ enrichment but prior to DEPArray™ single‐cell isolation. To allow storage at −20 °C and also avoid cellular damage due to freezing, we tested storing samples in high concentration glycerol at −20 °C. Initial experiments used the cell line LS174‐T spiked into HNV blood (1000 cells·mL^−1^ blood) which were then enriched using the CellSearch^®^ platform. Following enrichment, all cells were harvested from the CellSearch^®^ MagNest™ and half of the cells immediately isolated on the DEPArray^®^ (Fig. 3A). The remaining cells were suspended in glycerol and transferred to −20 °C for 24 months prior to DEPArray™ isolation (Fig. 3B). Individual isolated LS‐174 cells underwent ampli1™ WGA and the resulting products were subjected to GII‐PCR to determine the GII of the cell line cells isolated pre‐ and postglycerol storage (Fig. 3A,B). No significant difference in the GII of the individual recovered cells pre‐ and postglycerol storage was seen (Fig. 3C), indicating long‐term storage of CellSearch^®^‐enriched samples in glycerol has no detrimental effect on the DNA integrity of the cells.

**Figure 4:**
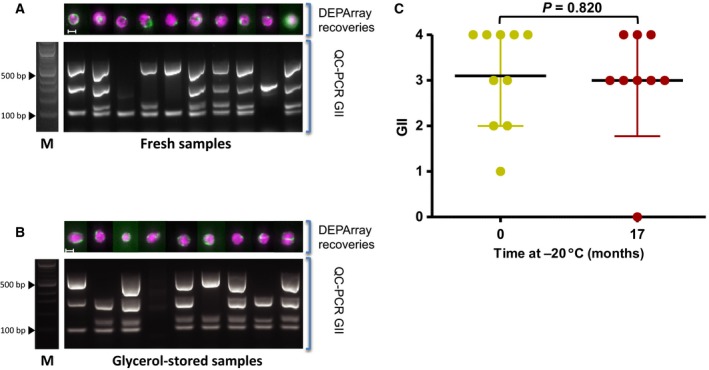
(A) CellSearch^®^ was used to enrich CTCs from a SCLC patient blood sample and DEPArray™ was used to isolate CTCs from a portion of the enriched sample prior to storage in glycerol at −20 °C. All isolated cells were subjected to WGA plus GII‐PCR and data presented using the same format as in Fig. 2 panel A. (B) An aliquot of the same CellSearch^®^‐enriched cells described in panel A was stored in glycerol at −20 °C for 17 months prior to CTC isolation by DEPArray™. All isolated cells were subjected to WGA plus GII‐PCR and data presented using the same format as in Fig. 2 panel A. (C) A comparison of the GII from cells enriched by CellSearch^®^ and DEPArray™ isolated prior to glycerol storage (0 months), and stored at −20 °C for 17 months in glycerol prior to DEPArray™ isolation. No statistically significant difference in the GII values was found following long‐term storage (Mann–Whitney test; *P* = 0.820; error bars show SD).

**Figure 5:**
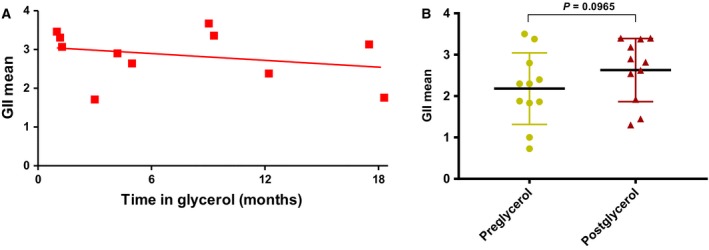
(A) The average GII for 11 clinical samples was established for CellSearch^®^‐enriched samples following glycerol storage at −20 °C for up to 18 months. Following DEPArray™ isolation, cells were subjected to WGA and GII‐PCR. Linear regression analysis was applied and showed no detrimental effect of long‐term storage shown by the trend line (red line) (*P* = 0.4056; *R* square = 0.078). (B) A comparison of the average GII of CellSearch^®^ cells isolated by DEPArray™ pre‐ and postglycerol storage at −20 °C. Following DEPArray™ isolation, each CTC was subjected to WGA and QC‐PCR to determine GII. The GII mean was not statistically significantly improved in the group of samples stored in glycerol at −20 °C.

Next, we tested long‐term glycerol storage of clinical samples. To this end, we collected CTCs from patients with SCLC by CellSearch^®^ and following harvesting, aliquots of each enriched sample were assessed immediately using DEPArray™ for single CTC isolation, with the remainder of the sample being stored in glycerol at −20 °C. For the first patient, the glycerol sample was stored at −20 °C for 17 months prior to DEPArray™ isolation (Patient 23; Table 2) and there was no observable or statistically significant difference seen for postglycerol‐stored cells when compared to cells isolated immediately after CellSearch^®^ enrichment (Fig. 4C). To confirm these initial findings, we expanded our analysis to an additional 10 patients covering three cancer types (SCLC, NSCLC, and prostate cancer) (Table 2). For all patient samples, CTCs were enriched by CellSearch^®^ with one aliquot of the enriched sample ran immediately on the DEPArray™ and the remaining sample stored in glycerol at −20 °C for up to 18 months prior to DEPArray™ isolation (Table 2). Analysis of the samples showed that the mean GII postglycerol storage remained high across all storage time points, up to 18 months post‐CellSearch^®^ enrichment (Fig. 5A). Pairwise analysis of the samples pre‐ and postglycerol storage showed no detrimental effect of storage across all 11 patient samples with the postglycerol samples showing a slight but statistically insignificant improvement in DNA integrity (Fig. 5B). Analysis of the effect of storage at set time intervals of 0–6 months, 6–12 months and 12–18 months found that there was no statistically significant change in the mean GII of all CTCs in clinical samples across all time points (Fig. S1).

**Table 2:** Details of the 11 clinical samples evaluated pre and post‐glycerol storage. Details include: type of disease; the number of CTCs; the total cell count after CellSearch^®^ enumeration; the predicted DEPArray counts (based on volumes transferred and a 30% of dead volume in the DEPArray cartridge); actual DEPArray total cell counts; actual DEPArray CTC counts (DAPI+/CK+/CD45−) and the time in months of glycerol storage at −20 °C

Patient	Disease type	CellSearch® count		Predicted DEPArray™ count based on volumes applied		Actual pre‐glycerol DEPArray™ count		Actual post‐glycerol DEPArray™ count		
		CellSearch® CTC count	Total cell count	CTC count per aliquot	Total cell count	CTC count	Total cell count	Time in glycerol (months)	CTC count	Total cell count
23	SCLC	312	514	109	180	31	148	17.0	120	252
24	SCLC	1522	2009	533	703	247	280	9.0	267	631
25	SCLC	1612	1949	564	682	425	1355	24.0	97	170
26	NSCLC	896	9095	209	2122	54	739	4.0	199	1487
27	NSCLC	471	1785	110	417	66	1242	12.0	96	3344
28	NSCLC	342	827	80	193	43	167	9.0	100	197
29	NSCLC	582	3308	136	772	138	1664	1.3	93	1180
30	NSCLC	634	8324	222	2913	315	13 222	18.0	197	11 273
31	NSCLC	175	5205	61	1822	232	11 254	5.0	144	10 122
32	NSCLC	117	735	41	257	39	247	1.0	31	324
33	Prostate adenocarcinoma	1032	8606	241	2008	358	814	0.1	199	425

SCLC, small cell lung cancer; NSCLC, non small cell lung cancer.

**Figure 6:**
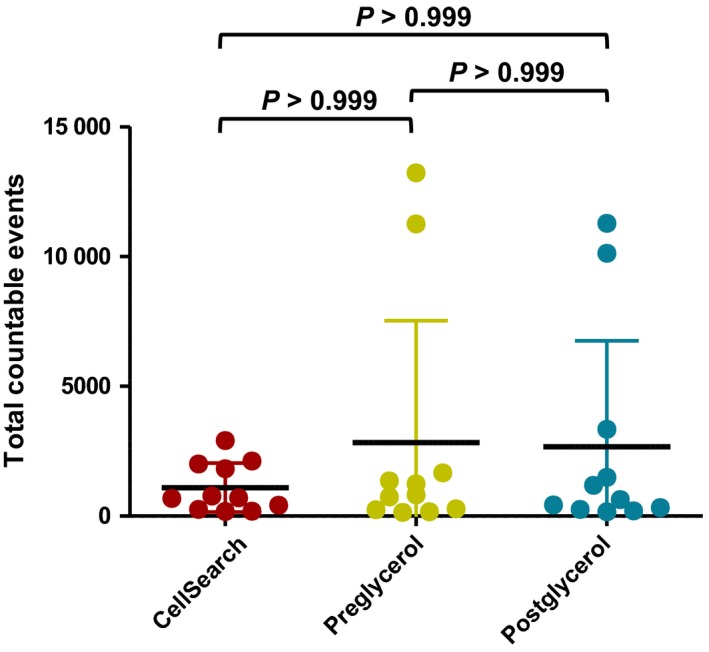
Total countable events from CellSearch^®^ were compared to the number of total events assessed by DEPArray™, pre‐ and postglycerol storage at −20 °C. No statistically significant difference was seen following analysis of paired samples (Friedman's test with Dunn's correction; error bars show SD).

To address whether glycerol storage could have a detrimental effect on total cell numbers, we compared the total cell numbers for all 11 clinical samples following CellSearch^®^ enumeration pre‐ and postglycerol storage. No significant differences in total cell counts were observed between the CellSearch^®^ and either pre‐ or postglycerol storage results (Friedman's test with Dunn's correction, *P* > 0.05) (Fig. 6).

## 4 Discussion

Both CTCs and circulating free DNA (cfDNA) can be readily obtained from a simple peripheral blood draw and together represent a ‘liquid biopsy’ that holds considerable potential for providing biomarkers that can improve cancer patient management (Carter *et al*., 2017; Diaz and Bardelli, 2014; Fusi *et al*., 2013; Krebs *et al*., 2014; Rothwell *et al*., 2016). The isolation and characterization of single tumour cells from the circulation can give an insight into the heterogeneity and genetic status of tumours, which has been associated with prognosis (Carter *et al*., 2017; Casasent *et al*., 2017; Navin, 2015). However, despite the promising clinical significance, the costs associated with isolation and molecular characterization of single CTCs is a significant obstacle. Consequently, preisolation storage would be advantageous, enabling careful selection of the samples to be subsequently analysed. Our aim was to establish a workflow that could be readily implemented and would allow the long‐term storage of enriched CTCs samples at −20 °C in glycerol.

The CellSearch^®^ semiautomated CTC enrichment and staining system has been established as a reliable process that has been adopted worldwide and CellSearch^®^ CTC counts have been recognized by the FDA as prognostic in a number of cancers. Although CellSearch^®^ CTCs can be readily isolated after enrichment, the process of analysing a single CellSearch^®^ patient sample which may contain many CTCs is both time‐consuming and costly so we have evaluated CTC ‘stop‐off’ points where CTCs can be banked, then retrieved for analysis. Our initial experiments focused on the stability of single CTCs isolated by combined CellSearch^®^ and DEPArray™ (Figs 2, 3, 4). Storage of isolated single cells from clinical samples at −80 °C showed that there was no measurable decline in genome integrity for at least 37 months. These results demonstrate that storage of isolated cells at −80 °C provides long‐term stability for subsequent genomic analysis.

Having established that isolated cells can be stored for long periods at −80 °C, we next looked for approaches that can be used to bank CellSearch^®^‐enriched cells prior to single‐cell isolation. For this, we identified glycerol as a potential cryoprotective agent since it has been used in high concentrations (40–50%) for blood cells storage in clinical practice (Henkelman *et al*., 2015; Morris *et al*., 2006; Ragoonanan *et al*., 2013) and it may be a superior alternative to other cryoprotectants, such as DMSO, which may adversely affect cells by exposure to strong bipolar compounds (Best, 2015; Henkelman *et al*., 2015). We first examined storage in glycerol at −20 °C using cells from a cell line ‘spiked’ into HNV blood collected using CellSave^®^ blood collection tubes, then enriched via CellSearch^®^ and subsequently stored in glycerol at −20 °C for 24 months prior to DEPArray™ isolation. Data from this ‘spike‐in’ experiment showed there was no statistical difference observed based on the GII of the recovered cells pre‐ and postglycerol storage (Fig. 3C). Although these ‘spike‐in’ experiments were encouraging, we were aware of the limitations of using cell lines due to the potential intrinsic differences, both biological and physical between cell lines and true CTCs. To confirm the clinical utility of our approach, we extended the glycerol storage at −20 °C to 11 clinical samples from three disease types and again compared the GII pre‐ and postglycerol storage at −20 °C. The results from the clinical samples confirmed that glycerol storage did not have a detrimental effect on the GII of samples (Figs 4 and 5). Interestingly, paired analysis of the clinical samples showed a minor improvement in the GII of glycerol‐stored samples compared to freshly processed samples, although this was not statistically significant (Fig. 5B.). These results are from a limited cohort of three different cancers (SCLC, NSCLC, and prostate cancer), and the effect of storage of CTCs from other disease types may differ and is part of an ongoing study in the laboratory.

One concern raised by these results was that the storage of enriched samples may lead to the loss of potentially more ‘fragile’ cells, for example cells in the early stages of apoptosis, with only ‘robust’ cells surviving long‐term storage, which was then reflected in apparent improved GII values. This bias could be problematic as the number of CTCs in clinical samples is usually low and the loss of cells would decrease our ability to analyse patient samples effectively. However, both the total number of cells and the total number of CTCs were found to be unchanged following glycerol storage, demonstrating that glycerol storage does not lead to cell loss (Fig. 6). Another possible explanation for the improved GII of the glycerol samples is that although the cells enriched by CellSearch^®^ are fixed, it has been shown that glycerol has a cryoprotective effect on cells (Ragoonanan *et al*., 2013). This additional cryoprotection may protect the cells during DEPArray™ manipulation, increasing the integrity of the cell membranes and thereby maintaining the GII of these samples.

In conclusion, the results presented in this study indicate that CellSearch^®^‐enriched samples can routinely be stored in glycerol at −20 °C, prior to single‐cell isolation and molecular analysis. This approach greatly increases the utility of CTCs as a liquid biopsy in the clinical setting, where they have potential in addressing questions of tumour response and heterogeneity, as well as giving insight into mechanisms of resistance due to the simplicity of longitudinal collection of bloods.

## Author contributions

GB, CD and DGR designed and supervised the project. BM, DGR and GB co‐wrote the manuscript, planned, ran the experiments and analysed the data. DB ran all the DEPArray™ isolations. FC, JA and LC provided additional experimental input. LC, FHB and LK provided clinical input including establishing ethical permission and patient consent for blood samples. All authors read and approved the final manuscript.

## Supporting information


**Fig. S1.** The graph shows pre and post‐glycerol storage GII data from 11 clinical samples divided into 3 groups based the length of time samples were stored in glycerol at −20 °C.
