# Supplementary figures and images for: Molecular analysis of single circulating tumour cells following long‐term storage of clinical samples
Source: Mol Oncol. 2017 Oct 24;11(12):1687–97. doi: 10.1002/1878-0261.12113 (PMC5709616; doi:10.1002/1878-0261.12113)

Supplementary Figure S1

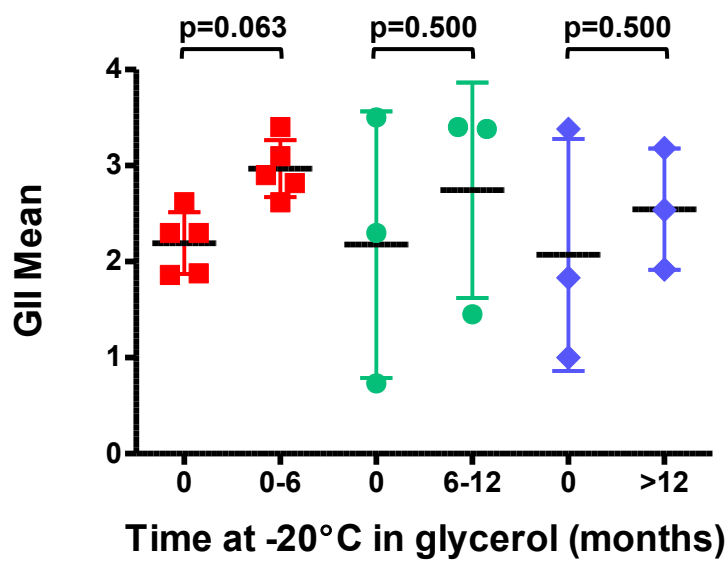

Supplement: Supplementary file 1 — Fig. S1. The graph shows pre and post‐glycerol storage GII data from 11 clinical samples divided into 3 groups based the length of time samples were stored in glycerol at −20 °C. [file MOL2-11-1687-s001.pdf]
